# Prevalence and factors associated with transmission of lymphatic filariasis in South Sudan: a cross-sectional quantitative study

**DOI:** 10.11604/pamj.supp.2022.42.1.33895

**Published:** 2022-06-10

**Authors:** Mutale Nsakashalo Senkwe, Kibebu Kinfu Berta, Samuel Makoy Yibi Logora, Julia Sube, Alex Bidali, Abias Abe, Adiele Onyeze, Jane Pita, John Rumunu, Sylvester Maleghemi, Fabian Ndenzako, Olushayo Oluseun Olu

**Affiliations:** 1 World Health Organization, WHO Country Office, Juba, South Sudan,; 2 Ministry of Health, Juba, South Sudan,; 3 National Public Health Laboratory, Juba, South Sudan,; 4 Multicountry Assignment Team Support Team, World Health Organization, Nairobi, Kenya

**Keywords:** Lymphatic filariasis, circulating filarial antigen, immunochromatographic test, filarial test strip, mapping surveys, South Sudan

## Abstract

**Introduction:**

South Sudan is affected by a high burden of Neglected Tropical Diseases (NTDs). The country is very vulnerable to NTDs due to its favourable tropical climate and multiple risk factors. However, the distribution of the diseases and the populations at risk for the various NTDs is unknown. This paper describes the distribution of lymphatic filariasis (LF) in 58 counties of South Sudan.

**Methods:**

a descriptive quantitative cross-sectional study of LF in 58 counties in 8 states of South Sudan recruited adult volunteers aged ≥ 15 years tested for circulating filarial antigens (CFA). A quantitative descriptive statistical was performed to determine the prevalence rates and the endemicity (CFA positivity rate ≥1%) of lymphatic filariasis in 9213 adult individuals from 101 villages.

**Results:**

the overall prevalence of positive CFA was 1.6%, and the highest state prevalence was reported in the Upper Nile state at 3.4%. Based on the prevalence of positive CFA 64% of the surveyed counties are endemic to lymphatic filariasis. The endemicity ranged from 1-11.1% positive CFA. The highest prevalence of positive CAF was observed in the >50 years old age group (2.7%), followed by the 46-50 age group (2.3%). Males tested more positive than females (52.4% Vs 47.6%). Participants were three times more likely to test positive for CFA on filarial test strips (FTS) compared to immunochromatographic test (ICT). There was a statistically significant difference in the prevalence of positive CFA among the two tests (P=.002).

**Conclusion:**

the distribution of LF is widespread, with varying transmission risks. The produced prevalence maps of infection provided evidence on the areas for targeted interventions in the national NTD program in South Sudan. An increased number of positive CFA were identified using FTS than ICT; hence, it is advisable to use FTS in the future transmission survey.

## Introduction

Lymphatic filariasis (LF), also known as elephantiasis, is a vector-borne parasitic disease caused by filarial parasites *Wuchereria bancrofti, Brugia malayi*, and *B. timori*, which are transmitted from person-to-person by mosquitos in the genera Culex, Anopheles, Mansonia, and Aedes [1-3]. Lymphatic filariasis is one of the preventive chemotherapy Neglected Tropical Diseases (NTDs) [4], which is mainly endemic in the tropics and subtropical areas primarily affecting the poor and marginalized communities. Globally, it is estimated that *Wuchereria bancrofti* causes 91% of LF cases [5]. Once a person is infected, the parasites nest in the lymphatic vessels causing damage, which leads to lymphoedema, elephantiasis of limbs, and hydroceles [6]. The affected persons are often subjected to stigmatization and discrimination [7]. Most infected people do not show any signs or may present with acute filarial episodes. Notably, the risk of developing clinical manifestations decreases with mass drug administration of either ivermectin or diethylcarbamazine in combination with albendazole [5]. An estimated 856 million people who live in 72 endemic countries are at risk of LF, out of which120 million are estimated to be infected with the disease.

Known risk factors for LF include age, sex, non-utilization of insecticide-treated bed nets (ITN), occupation-dependent exposure to mosquitoes such as in farmers, hunters, and source of water [8,9]. South Sudan is very vulnerable to transmission of LF due to high levels of poverty, low literacy rates (27%), and household clustering in the rural, remote and peri-urban settings [10]. These are compounded by an increased likelihood of extreme climatic events such as floods, high temperatures, and moisture conditions, particularly in swampy areas along the Nile River [11]. Information and data on LF in South Sudan are scarce. Anecdotal information suggests that LF may be endemic in all 10 states; however, existing data indicate LF is endemic in three States (i.e. Western Equatoria, Central Equatoria, and parts of East Equatoria) and non-endemic in Northern Bahr el Ghazal and Unity states [12,13]. Although these observations suggested that transmission of LF is ongoing, the actual geographical distribution, extent, and LF prevalence across the country remain unknown. As a response to the resolution at the World Health Assembly (WHA) of 1997 to eliminate LF globally and the control and elimination milestones and target in the WHO 2021-2030 NTD Roadmap, it is imperative to understand the burden of LF in the country for targeted and scaled up interventions [14,15]. Therefore, this study aimed to provide empirical information on the LF prevalence and risk factors associated with LF transmission in South Sudan as observed from LF mapping surveys conducted using Immunochromatographic Test (ICT) and Filarial Test Strip (FTS).

## Methods

**Study design and area:** we conducted a cross-sectional quantitative study using the WHO survey guideline for LF in adults ≥ 15 years of age in South Sudan from 2016 and 2019 [16]. South Sudan is administratively divided into 10 states and three administrative areas, which are further divided into 80 counties, of which 58 had no reliable prevalence data. Due to the prevailing insecurity, the study was conducted in two phases in 2016, phase 1 covering 26 counties and phase 2 in 2019, covering 32 counties.

**Study site selection and sample size:** a three-stage cluster sampling method drawn from the WHO’s Rapid Assessment for Geographical Distribution of Lymphatic Filariasis (RAGFIL) was used [17]. Twenty-two counties, five in Northern Bahr El Ghazal in 2009 (14), eight in Unity, three in Eastern Equatoria, and six in the Central Equatoria States in 2010, with recent LF prevalence data, were identified and excluded from the current study [13]. The 52 counties in the six remaining states, plus Panyijar county from Unity and Kapoeta South, Kapoeta North, Torit, Magwi, and Lapon counties from Eastern Equatoria were included in the study.

**The next stage identified the Payams:** a Payam is the second-lowest administrative division next to the county using a simple random selection of two villages at least 50 km buffer zone because evidence has shown filariasis foci to be homogenous within a 50 km diameter [18]. Thus, 119 study sites were selected using the lot quality assurance sampling (LQAS) method for a more homogenous population [19]. Individuals who had lived in the village for more than 10 years were selected and tested for W. bancrofti circulating filarial antigen (CFA). The risk of exposure to mosquitoes (host-vector) increased with the number of years resided in the LF endemic areas; hence, WHO guideline-recommended as selection criteria [16]. Exposure to filarial Testing was stopped if two or more people tested positive within each selected village among the first 50 individuals (balanced by gender). Otherwise, testing continued up to 100 individuals. The team sampled adjacent villages if they could not reach this target.

Two key test types were used to rapidly diagnose bancroftian filariasis and its distribution in areas with persistent infections, in 2016 BinaxNOW® Filariasis card test (immunochromatographic card test (ICT) was used, and the Alere Filarial Test Strip (FTS) in 2019. Both Alere FTS and ICT test cards are qualitative point-of-care diagnostic tools that detect *W.bancrofti* CFA in human blood, plasma, or serum [20]. The ICT card test has been used in the Global Program to Eliminate Lymphatic Filariasis (GPELF) since 2000, while the FTS was introduced in 2013 [21]. In both tests, counties were considered endemic if the CFA positivity rate was > 1.0%.

**Data collection:** nine teams were composed, each comprising a supervisor (i.e. laboratory technologist or an experienced laboratory technician), two laboratory technicians, a data clerk and a social mobilizer. Local health personnel led the study teams to the study areas and assembled community members at either a clinic or health centre for the test, which was convenient for the purpose. Geographical coordinates at each site were taken using smartphones. One millilitre (ml) finger-prick blood was collected from eligible individuals using a heparinized capillary tube and tested for CFA using a rapid ICT card (ICT card, Binax Inc., USA) or the filarial test strip (Alere Filariasis Test Strip). The location, date, name, sex, age and results were entered on the study form on the phone and backed up on a hard copy.

**Data analysis:** data was captured on Bold Like Us (BLU) studio 5.5 smartphones running Android 4.2 (Jelly Bean) through a modified version of Open Data Kit (ODK) with LINKS application with a server hosted at the national level. The questionnaires and forms collected using ODK in the field were transferred to an aggregate server situated at the national level. Once in the server, an ODK briefcase was used to download data using an export function from the ODK aggregate server (ona.io) while connected to the internet. The questionnaires were downloaded from the Briefcase into Microsoft Excel format for data cleaning and analysis. We conducted a descriptive analysis using IBM Statistical Package for Social Science for Window Version 26.0 (IBM SPSS V26) and produced maps using ArcGIS (ESRI, California, and the USA). The descriptive analysis included socio-demographic characteristics and epidemiological distribution of LF, determination of the proportion of the test result by sex, age group, insecticide-treated bed nets, and test type. For continuous variables like age, we computed mean, median, mode, range and standard deviation; while, for categorical variables such as state, county, gender, age group, and test type, we ran frequency distribution. A two-by-two table was used to determine the relationship between tests types (FTs versus ICT) and test results (positive versus negative), including odds ratio and Pearson´s Chi-Square test. A 95% confidence interval (CI) at α significant level 0.05 was used to reject or accept the null hypothesis based on the calculated significance level and odds ratio.

**Ethical consideration:** during the survey in 2016/2018 ethical approvals was obtained from the Research and Ethics Committee of the national Ministry of Health. Consent was obtained from all persons examined. All positive cases found during the study were treated with ivermectin 150-200 microgram per kilogram of body weight combined with albendazole 400 milligrams. In the manuscript, secondary data was used with anonymity without ethical clearance.

## Results

Study site, study population and prevalence of CFA: a total of 9213 individuals from 101 survey sites located in 58 counties in eight States of South Sudan were tested for CFA and had valid results indicated in Table 1. The age of tested individuals ranged from 15 to 100 years. The mean age was 37 years, with a standard deviation of 14.8 years. The age group that participated the most was 26-30 years (16.8%), followed by those above 50 years (15.9%), who also contributed the highest proportion of positive results (26.3%) (Table 1). Males tested more positive than females (52.4% Vs 47.6%), but there was no statistically significant difference among gender. Participants were three times more likely to test positive for CFA on filarial test strips (FTS) compared to immunochromatographic test (ICT) (2.2% Vs 0.9%). There was a statistically significant difference in the prevalence of positive CFA among the two tests (P=.002) (Table 2).

**Table 1:** socio-demographic characteristics and epidemiological distribution of the prevalence of positive circulating lymphatic filariasis in South Sudan - 2016 and 2019

Variable	Category	The total number (%) tested positive	The total number (%) tested negative	Total number (%) of test
**Age group**	15-20	14(1.1%)	1291(98.9%)	1305(14.2%)
	21-25	3(0.30%)	913(99.7%)	916(9.9%)
	26-30	15(1.0%)	1531(99.0%)	1546(16.8%)
	31-35	22(2.1%)	1017(97.9%)	1039(11.3%)
	36-40	24(1.9%)	1234(98.1%)	1258(13.7%)
	41-45	11(1.3%)	862(98.7%)	873(9.5%)
	46-50	19(2.3%)	794(97.7%)	813(8.8%)
	>50	39(2.7%)	1424(97.3%)	1463(15.9%)
**Sex**	Female	70(1.4%)	4817(98.6%)	4887(53.0%)
	Male	77(1.8%)	4249(98.2%)	4326(47.0%)
**State**	Eastern Equatoria	10(2.1%)	463(97.9%)	473(5.1%)
	Jonglei	20(0.9%)	2137(99.1%)	2157(23.4%)
	Lakes	7(0.5%)	1395(99.5%)	1402(15.2%)
	Unity	0(0%)	197(100%)	197(2.1%)
	Upper Nile	53(3.4%)	1535(96.6%)	1578(17.1%)
	Warrap	8(0.6%)	1273(99.4%)	1281(13.9%)
	Western Bahr El Ghazal	2(0.3%)	589(99.7%)	591(6.4%)
	Western Equatoria	47(3.1%)	1487(96.9%)	1534(16.7%)
	Total	147 (1.6%)	9076 (98.4%)	9213(100%)

**Table 2:** prevalence of positive circulating lymphatic filariasis antigen in South Sudan - 2016 and 2019

Parameters	Positive	Negative	Total	Odds ratio	P-value	95% confidence interval
Male	78	4248	4326	1.28	0.135	0.92, 1.77
Female	69	4818	4887			
Total	147	9066	8213			
Filarial test strips	113	5133	5246	3.18	0.002	1.46, 6.93
Immunochromatographic test	34	3933	3967			
Total	147	9066	9213			

**Figure 1:**
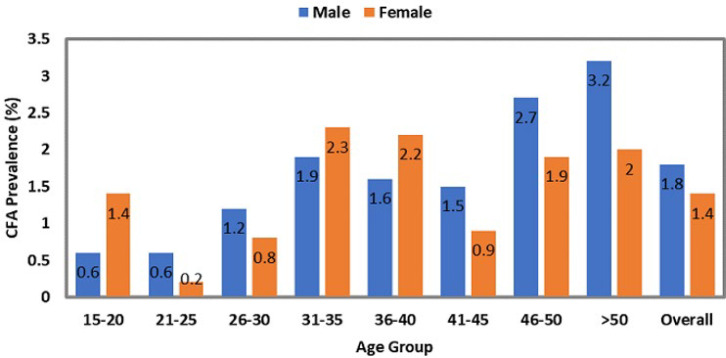
circulating filarial antigen prevalence by sex and age group: study findings of the fieldwork conducted in South Sudan - 2016 and 2019

**Figure 2:**
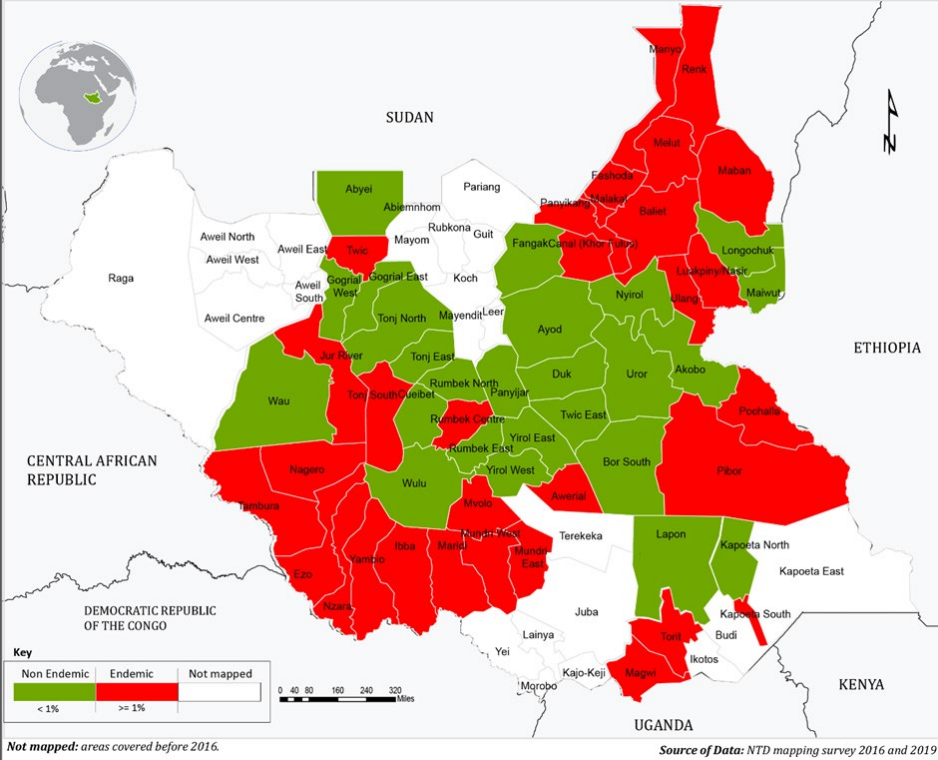
lymphatic filariasis endemicity map South Sudan: study findings of the fieldwork conducted in South Sudan in 2016 and 2019

The highest number of individuals tested was reported in Jonglei at 23.4 % (2157/9213), followed by Upper Nile (17.1%), Western Equatoria (16.7 %), and Lakes (15.2 %) (Table 1). The overall prevalence of positive CFA was 1.6% representing 1.8% in males and 1.4% in females (Figure 1) and was observed more in the >50 years old age group (2.7%), followed by the 46-50 age group (2.3%). At state level the proportion ranged from 0 to 3.4% (Table 1). The highest proportion of positive cases was seen in Upper Nile (36.1%), Western Equatoria (32.0 %), and Jonglei (13.6%). Out of the 58 counties, 37 (63.7%) were endemic for LF (Figure 2).

All counties in Western Equatoria state endemic while all except one county in Eastern Equatoria (Kapoeta North) and two in Upper Nile (Longochuk and Maiwut) were endemic for LF. The prevalence of positive CFA varied from 0%-11.1% by county with highest being Pibor 11.1%, Renk 9.5%, Tambura 9.2%, Melut 7.2%, Maban 7.2%, Kapoeta South 6.2%, and Khorflus 6.1% (Figure 2). A total of 7,667 (83.2%) surveyed participants resided in the altitude range of 389 meters to 560 meters above sea level, while the remaining 1,551 (16.8%) lived above 560 meters. The prevalence of positive CFA was 1.5% (113/7662) versus 2.2 (34/1551) respectively.

## Discussion

This study presents the results of the first community-wide study to elucidate LF prevalence in eight states of South Sudan. The findings show the prevalence of W. bancrofti in the majority of the 58 counties sampled, which confirms the anecdotal evidence that LF is prevalent in all the 10 states of the country [22]. The ongoing transmission is widespread and conforms to previous predictions of LF in South Sudan (21); however, in many of the counties, the prevalence was low. A high prevalence of positive CFA was observed in the areas near national borders in the North-eastern part of the country (i.e. Upper Nile and the Jonglei States) and Western Equatoria State. Upper Nile falls within a region habitually known to be highly endemic. This finding is not surprising as the Blue Nile and the South Kordofan Sudan, which borders the Upper Nile, have a widespread high LF prevalence of more than 50% [23]. At the same time, Jonglei is close to the hyperendemic regions of Gambella and Beni Shangul Gumuz of Ethiopia [24]. While Western Equatoria State borders the highly endemic area of the neighbouring Democratic Republic of Congo (DRC) [25]; an occurrence that could explain the increased prevalence of LF in the counties of the western equatorial state bordering endemic areas of DRC. It is not clear why most of the central part of the country is non-endemic for the disease; perhaps this could be attributed to a less conducive environment for vector breeding which drives the transmission of W. bancrofti. This finding thus needs to be assessed further, including the persistently high prevalence in the other areas.

Both the FTS test and (ICT) test card are qualitative point-of-care diagnostic tools that detect W. bancrofti CFA in human blood, plasma, or serum. The ICT card test has been used in the global program to eliminate lymphatic Filariasis (GPELF) since 2000, while the FTS was introduced in 2013 [21]. Since its introduction FTS test had shown superiority over the ICT card test in terms of both sensitivity and specificity. The study reaffirmed the higher sensitivity and specificity of the FTS test compared ICT card test [20,26]. These findings are similar to a study conducted in Liberia where the detection rate was 26.5% more with FTS than ICT [20,27]. Furthermore, ICT loses its sensitivity in low endemic areas, particularly in individuals exhibiting low CFA levels [26,27]. This raised serious concerns, especially in the counties that were mapped lymphatic filariasis CFA in 2016 using ICT. Due to the significant difference in test sensitivity between the test types used, some of the villages found to be non-endemic could be endemic. Therefore, the national LF programme should implement a more sensitive test to monitor and evaluate the impact of the interventions is highly recommended [28].

An interesting finding from this study is the non-statistically significant higher CFA positivity in males versus females. In most instances, it is believed that males go out to work in the field and have a higher chance of exposure to the bite of parasite carrying mosquito, which shows an increasingly higher infection than females [29]. Moreover, the compelling evidence that women of the reproductive age group bear immunity to LF infection adds to the lower number of positive results observed in females [30,31]. An in-depth analysis of sex differentials in prevalence, density and clinical pathology study revealed a higher prevalence of infection in males than females. However, in South Sudan, several factors equally affect males and females for mosquito bites such as flooding, displacement, and poor socio-economic status [22,32]. These exposures may explain an increased prevalence of LF in males and females without significant sex differentials. Hence, this finding is critical in the elimination programme, ensuring a high treatment coverage in males and females to interrupt transmission.

The older age group mainly affected by LF in South Sudan is consistent with other similar prevalence studies [31]. This can have implications during impact assessment as the focus is on young children for new infections. Inclusion of the older age group in the evaluation may accurately represent the presence or absence of infection. Other studies have shown increasing microfilaria rate in children until the age of 20-30 years, then it remains constant or decreases due to host immunity [6,30]; an observation was also made in our study.

Environmental factors play a critical role in the transmission of LF in communities [33]. High altitude is protective in humans for LF infection due to the constant negative association between vector breeding capacity and increases in altitude as it becomes less suitable for parasite survival. The prevalence of LF in our study is higher at an altitude above 560 meters than an altitude range of 389 to 560 meters (70.7% versus 29.3%) [23]. Of the total surveyed participants, 73% of the surveyed participants resided in the altitude range of 389 to 560 meters above sea level, while the remaining 27% lived above 560 meters. The altitude for South Sudan is low in most parts of the country with the highest altitude of the sites recording positive results in our study was at 780 meters. This is within the favourable altitudes in other countries, ranging from 100 meters to 1600 meters. Hence, the areas included in our study are within the favourable altitude of mosquito breeding and LF transmission.

A significant decline in LF prevalence observed in areas with effective implementation of vector control measures even before community-based mass drug treatments for LF commences assists in controlling and eliminating LF [34]. In most African countries, the malaria vector species also tend to be the principal LF vectors. Where vector control measures are scaled up and sustained, especially under the malaria control programme, there are secondary benefits to the LF programme as a reduction in CFA prevalence is observed [1]. The experts’ review of LF elimination highlighted the importance of integrating vector control under malaria control for this purpose [3,5,35]. In 2009, South Sudan scaled up the distribution of ITN as a vector control measure against malaria infection [36]. However, the number of cases continued to increase due to inadequate utilization of the nets and made the population is at very high risk of malaria and LF.

## Conclusion

The persistent high prevalence of CFA in South Sudan requires a more substantial move towards an integrated strategy that includes vector control, advocacy and social, behavioural change communication [35,37]. Still, the inadequate data on the exact vector(s) that transmits LF and their distribution across the country may limit the cause-effect and the association to specific vector control measures. The present study has provided new data on the epidemiology of lymphatic filariasis in South Sudan. This new knowledge is useful in the implementation of the GPELF goals which are aimed at stopping the spread of disease and the management of morbidity. In light of the high prevalence of positive CFA in the Upper Nile, Jonglei and Western Equatoria state that share borders with Sudan, Ethiopia and DRC, respectively, the country requires concerted efforts and effective policy to interrupt LF transmission in these areas with a focus on cross-border coordination and synchronization of LF preventive and control interventions. The superior sensitivity of FTS to ICT shown by this study provides evidence for policy recommendations on the type of tests to use for transmission assessment surveys to evaluate the success of LF elimination after mass drug administrations in South Sudan and other similar contexts where more sensitive tests are required. Although our study has generated evidence for a national LF elimination programme, useful information to further improve programme implementation is required. The GPELF recommends mass drug administration (MDA), morbidity management and disability prevention (MMDP) as strategies to achieve LF preventive goals [38]. Our study did not include the search for LF morbidity such as hydrocele, lymphoedema in those who participated in the study; hence, future research should estimate LF morbidity in the country. The NTD programme in the Ministry of Health should also prioritize LF morbidity assessment at every available opportunity. Integrating active case search for hydrocele and lymphedema during the mass drug administration (MDA) and other public health interventions is an option in this regard. Besides, there are still grey areas that require further research, such as the low positive correlation between the LF prevalence and altitude, particularly at the actual site and not at the county level.

### What is known about this topic

- Although lymphatic filariasis is endemic in South Sudan, the geographic distribution of the disease is unclear;
- While the general risk factors for transmission of the disease is known, these factors remain unclear in the country;
- The superior sensitivity of filarial test strips over immunochromatographic test in the detection of circulating filarial antigens.

### What this study adds

- This study provides information on the prevalence, risk factors, and distribution of in South Sudan;
- Key recommendations for scaling up effective and integrated public health measures for prevention and control of lymphatic filariasis.
